# Backward Cherenkov radiation emitted by polariton solitons in a microcavity wire

**DOI:** 10.1038/s41467-017-01751-6

**Published:** 2017-11-16

**Authors:** D. V. Skryabin, Y. V. Kartashov, O. A. Egorov, M. Sich, J. K. Chana, L. E. Tapia Rodriguez, P. M. Walker, E. Clarke, B. Royall, M. S. Skolnick, D. N. Krizhanovskii

**Affiliations:** 1 Department of Physics, University of Bath, Bath, BA2 7AY UK; 2 Department of Nanophotonics and Metamaterials, ITMO University, 197101 St. Petersburg, Russia; 3 ICFO-Institut de Ciencies Fotoniques, The Barcelona Institute of Science and Technology, Castelldefels, 08860 Barcelona, Spain; 4 Institute of Spectroscopy, Russian Academy of Sciences, Troitsk, 142190 Moscow Region, Russia; 5 Technische Physik and Wilhelm-Conrad-Röntgen Research Center for Complex Material Systems, Universität Würzburg, Am Hubland, 97074 Würzburg, Germany; 6 Department of Physics and Astronomy, The University of Sheffield, Sheffield, S3 7RH UK; 7 Base4 Innovation Ltd, Cambridge, CB3 0FA UK; 8 EPSRC National Centre for III-V Technologies, The University of Sheffield, Sheffield, S1 4DE UK

## Abstract

Exciton-polaritons in semiconductor microcavities form a highly nonlinear platform to study a variety of effects interfacing optical, condensed matter, quantum and statistical physics. We show that the complex polariton patterns generated by picosecond pulses in microcavity wire waveguides can be understood as the Cherenkov radiation emitted by bright polariton solitons, which is enabled by the unique microcavity polariton dispersion, which has momentum intervals with positive and negative group velocities. Unlike in optical fibres and semiconductor waveguides, we observe that the microcavity wire Cherenkov radiation is predominantly emitted with negative group velocity and therefore propagates backwards relative to the propagation direction of the emitting soliton. We have developed a theory of the microcavity wire polariton solitons and of their Cherenkov radiation and conducted a series of experiments, where we have measured polariton-soliton pulse compression, pulse breaking and emission of the backward Cherenkov radiation.

## Abstract (web summary)

Cherenkov radiation is generated from fast electrons and its optical analogue plays an important role in many nonlinear optical effects. Here, Skryabin et al. demonstrate backward-propagating photonic Cherenkov radiation generated from solitons in a semiconductor exciton-polariton system.

## Introduction

It was in 1934 that P. Cherenkov discovered the radiation effect, which now bears his name. Later, I. Frank and I. Tamm developed a theory of the effect, which explained the origin of the Cherenkov radiation. In recognition of their achievements all three researchers were awarded the 1958 Nobel Prize in Physics. A decade later, Veselago^1^ predicted that materials with negative refractive index should demonstrate backward Cherenkov radiation. During the recent wave of research into the negative index and other metamaterials the backward (reversed) Cherenkov radiation and its close analogues were observed experimentally^2,3^ and extensively studied theoretically, see, e.g., refs ^4–6^. Backward Cherenkov radiation was also predicted to occur in photonic crystals^7^ and in the quantum analysis of this renowned effect^8^.

While the standard Cherenkov radiation is emitted by electrons moving with a velocity above the phase velocity of light in a medium, a purely optical analogue of the Cherenkov radiation has been found to play a paramount role in the spectral shaping of pulses propagating in optical fibres^9–11^, silicon waveguides^12–14^ and, more recently, in the frequency comb generation in microring resonators^15,16^. In the photonic Cherenkov-like effect, an optical quasi-soliton pulse, not an electron, serves as the radiation emitter. The Cherenkov radiation by multiple solitons in photonic crystal fibres is the key effect behind supercontinuum generation^9–11^ that was used to develop a new generation of light sources^17^ and to determine the absolute spectral location of the frequency comb lines critical for precision spectroscopy research^18^.

The nonlinearity-induced backward Cherenkov radiation by solitons has not been so far observed and reported and it is the main subject of this communication. However, other types of the nonlinear backward emission of optical signals have been studied since the 1960s and into the twenty-first century and should be mentioned here. These include, e.g., backward Raman scattering^19^, backward wave generation through Brillouin scattering^20,21^ and backward quasi-phase-matched second harmonic generation^22,23^.

With the recent development of nanophotonics, nonlinear effects in silicon, silicon nitride, gallium arsenide and other semiconductor and dielectric waveguides and microresonators are playing an increasingly important role for optical signal processing in chip-scale devices^12,13,24–26^. One category of these devices uses the so-called strong coupling regime between the photons confined in a two-dimensional (2D) microcavity formed by two distributed Bragg reflector (DBR) mirrors and the quantum well excitons leading to the formation of half-light half-matter quasi-particles—exciton-polaritons or simply polaritons^26^. Polaritons have revealed remarkably strong nonlinear interactions and many fascinating quantum effects, including Bose–Einstein condensation^27,28^, superfluidity^29^ and quantised vortices^30,31^. So-called bright dissipative polariton solitons have been reported^32–34^ in microcavities with short polariton lifetime using a continuous wave (CW) pump holding beam that provided additional gain in order to compensate for the loss.

Although research into polariton solitons has been very intense^35,36^, there have been no reports of Cherenkov radiation by bright polariton solitons to the best of our knowledge. Observation of Cherenkov radiation requires long polariton lifetimes, enabling propagation of polaritons and solitons over long distances without the holding beam. So far only studies of conservative dark solitons in propagating superfluids in planar microcavities have produced encouraging results^37,38^, which were subsequently debated^39–41^.

**Fig. 1:**
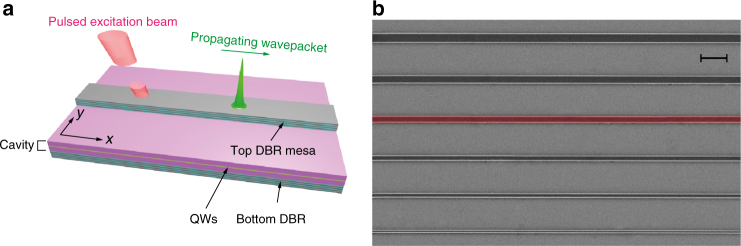
Microcavity wires (MCWs) and the excitation scheme. **a** A 3D schematic view of the system geometry. Top reflector is partially etched to form the polariton MCW. The pulsed excitation beam is applied at an angle along the *x*-axis, creating a polariton wavepacket with a controlled *x*-projection of the momentum. **b** An SEM image of the sample containing several MCWs of different widths. The 5 μm-wide wire studied experimentally is highlighted. Scale bar is 20 μm

In this work we present theoretical and experimental results demonstrating for the first time backward-propagating Cherenkov radiation. It is generated by bright conservative solitons in an exciton-polariton system. These results are made possible because of the long 30 ps polariton lifetime in microcavity wires (MCWs). The latter were fabricated as ridge waveguides formed by etching the surrounding material from the 2D microcavity to create additional confinement transverse to the propagation direction (Fig. 1). They are the natural choice for observation of solitonic effects since the additional confinement^42–47^ reduces the power degradation caused by defocusing in the transverse direction. In contrast to all previous studies of the Cherenkov radiation in optical fibres and semiconductor waveguides, where the radiation is always co-propagating with the emitting solitons^11–14^, the polariton Cherenkov radiation propagates in the direction opposite to that of the initial excitation and of the soliton. This unexpected and observationally dramatic effect arises from the unique dispersion of microcavity polaritons, which has intervals of positive and negative group velocities, with both normal and anomalous dispersions, within a narrow range of frequencies^42^. The intensity of the Cherenkov radiation is also boosted, compared to other nonlinear optical systems, by the extremely strong nonlinearity inherent to polariton systems^36,44^.

## Results

### Model equations

We start developing our theory of the backward polariton Cherenkov radiation by introducing the complex amplitudes *A*_±_ of the *σ*_+_ and *σ*_−_ polarised photonic microcavity modes coupled to the amplitudes of the wave functions of the positive and negative spin-one coherent excitons *ψ*_±_^47^. We account for the exciton-exciton interaction and for the spin-orbit coupling effects between the photonic components, originating from the TE/TM photon energy splitting^48^. Under these assumptions the resulting dimensionless equations are:^47,48^

1 $$i\partial _tA_ +	 + \left( {\partial _x^2 + \partial _y^2} \right)A_ + + \left( {i\gamma _c + \delta _c + \delta + U(y)} \right)A_ + \\ 	= - \psi _ + + \beta \left( {\partial _x - i\partial _y} \right)^2A_ -$$

2 $$i\partial _tA_ - 	 + \left( {\partial _x^2 + \partial _y^2} \right)A_ - + \left( {i\gamma _c + \delta _c + \delta + U(y)} \right)A_ - \\ 	= - \psi _ - + \beta \left( {\partial _x + i\partial _y} \right)^2A_ +$$

3 $$i\partial _t\psi _ + + \left( {i\gamma _e + \delta - \left| {\psi _ + } \right|^2 - g\left| {\psi _ - } \right|^2} \right)\psi _ + = - A_ +$$

4 $$i\partial _t\psi _ - + \left( {i\gamma _e + \delta - \left| {\psi _ - } \right|^2 - g\left| {\psi _ + } \right|^2} \right)\psi _ - = - A_ -$$

The photon, *γ*_*c*_, and the exciton, *γ*_*e*_, linewidth parameters are normalised to the Rabi splitting *ħ*Ω_*R*_ = 4.5 meV. For the polariton linewidth 0.1 meV, we have $\gamma _{c,e} \simeq 0.02$. *δ*_*c*_ = −0.4 and *δ* are the dimensionless detunings of the cavity resonant frequency and of the pulse carrier frequency from the exciton resonance, which is chosen by us as a reference. The above detunings are also normalised to *ħ*Ω_*R*_. $U(y) = e^{ - \left( {2y/w} \right)^8} - 1$ describes the lateral confinement in the MCW through total internal reflection, *w* = 7^47^. Spatial coordinates are normalised to the distance *L* = $\sqrt {\hbar {\mathrm{/}}\left( {2m_c\Omega _R} \right)}$, where $m_c \simeq 10^{ - 36}$ kg is the effective cavity photon mass, so that $L \simeq 0.8$ μm. One unit of the dimensionless time is $\Omega _R^{ - 1} \simeq 0.13$ ps. Spin-orbit coupling in the sample used in our experiments is two orders of magnitude smaller than the Rabi splitting. Its strength is characterised by the parameter *β*. We have estimated from the experimental data that the splitting of the transverse-electric (TM) and transverse-magnetic (TM) resonances splitting varies between 20 and 30 μeV, which is well approximated by taking *β* = 0.02. Cross-spin exciton-exciton interaction is introduced through the parameter *g* and is weakly attractive, *g* = −0.05^49^.

### Linear MCW polaritons

We start our analysis with calculation of the linear spectrum of a MCW. Disregarding the nonlinear terms and losses we set *A*_±_(*x*, *y*) = *a*_±_(*y*, *k*)*e* ^*ikx*^, *ψ*_±_(*x*, *y*) = *ψ*′_±_(*y*, *k*)*e* ^*ikx*^, where *k* is the momentum along the waveguide axis. The resulting linear eigenvalue problem for *a*_±_(*y*, *k*), *ψ*′_±_(*y*, *k*) and *δ*(*k*) is solved numerically.

**Fig. 2:**
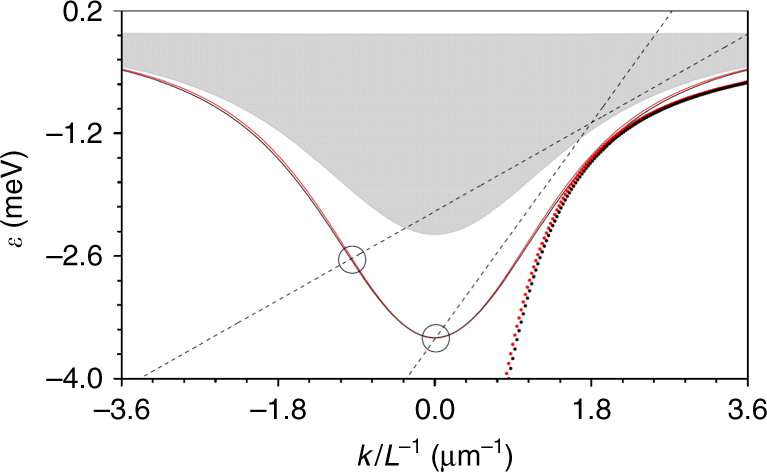
Polariton dispersion and Cherenkov resonances. Energy-momentum (or dispersion) characteristic of the lowest-energy modes is shown with solid red (TE mode) and black (TM mode) lines: *ε* = *ħω*_*R*_ *δ* vs. polariton momentum *k* *L* ^−1^. The approximate dispersion giving the exact solitons is shown with the dotted lines. The grey shaded area represents continuous spectrum. Two straight lines show examples of the dispersion characteristic of the exact solitons with *δ* = −0.35, *k*_s_ = 1.2, *v* = 0.366 (soliton spectrum centred at ≃2 μm^−1^, Cherenkov resonance at ≃0) and *δ* = −0.24, *k*_s_ = 1.5, *v* = 0.150 (soliton spectrum centred at ≃2.4 μm^−1^, Cherenkov resonance at ≃−0.9 μm^−1^). The open circles show the Cherenkov resonances, representing solutions of Eq. (12)

Figure 2 shows the corresponding energy vs. momentum (dispersion) plot for the MCW ground-state modes. Degeneracy between the TE and TM modes within the pairs is lifted through *β* ≠ 0. The effective polariton mass of the ground states $m_{{\mathrm{eff}}} = \hbar {\mathrm{/}}\left( {\Omega _RL^2\partial _k^2\varepsilon } \right)$ changes its sign from positive to negative at some critical momentum. In the optical waveguide terminology, it means that the group velocity dispersion changes from anomalous to normal and hence the dominantly repulsive exciton-exciton interaction can now result in the nonlinear self-focusing of polaritons.

The positive/negative momenta in our system are directly associated with the positive/negative group velocities (*∂*_*k*_ *δ*) and Poynting vectors: hence we are dealing with the usual positive index material and structure. A negative momentum wave generated by the positive momentum wavepacket through a nonlinear process, see below, constitutes the backward radiation in our terminology. As time evolves the forward and backward waves propagate in the opposite directions along the waveguide axis, *x*.

### Exact solitons and their stability

If a sufficiently intense polariton pulse is prepared in the ground state, and its momentum is high enough, then a large part of the pulse spectrum will belong to the negative mass range and the pulse itself is expected to demonstrate solitonic properties as it propagates down the MCW. This is due to the fact the pulse chirp induced by its kinetic spreading is compensated by the chirp induced by the polariton-polariton repulsion. However, a part of the spectrum unavoidably propagates under the conditions of positive effective mass. Therefore, some energy is expected to be emitted as dispersive Cherenkov radiation, which is well known in the context of fibre optics and, in particular, of supercontinuum generation in photonic crystal fibres^11,50^.

It is almost certainly impossible to find a natural system supporting exact soliton solutions under conditions where there is no continuous provision of energy into the soliton. Indeed, dissipation is always present in nature and the dispersion laws required for the existence of solitons are always idealised to some degree in mathematical models. In this regard, neither the polariton model, Eqs. (1)–(4), nor, for example, the generalised nonlinear Schrödinger equation used to model supercontinuum in photonic crystal fibres^11^ are exceptions. Therefore, understanding of the underlying physics of pulse propagation in our system can be facilitated if an approximate model with an exact soliton solution is introduced. After we establish such a model, we will use it to develop a theory of the dispersive wave emission by polariton solitons, adopting an approach similar to the one originally developed for fibre solitons^50^. A numerical modelling of the full two-dimensional Eqs. (1)–(4) and experimental results presented below verifies the validity of these approximations.

To find the exact solitons we first consider a one-dimensional approximation *∂*_*y*_ = 0. We introduce the soliton momentum *k*_s_ and proceed by substituting

5 $$A_ \pm = B_ \pm (t,x)e^{ik_{\rm s}x},\psi _ \pm = \phi _ \pm (t,x)e^{ik_{\rm s}x},$$

into Eqs. (1)–(4). We also assume that the spectra of the pulses under consideration are sufficiently narrow, so that we can disregard $\partial _x^2B_ \pm$ and *β∂*_*x*_ *B*_±_ terms in the first approximation. This leads us to

6 $$i\partial _tB_ + + i2k_{\rm s}\partial _xB_ + + \left( {\delta _c + \delta - k_{\rm s}^2} \right)B_ + = - \phi _ + - \beta k_{\rm s}^2B_ - ,$$

7 $$i\partial _tB_ - + i2k_{\rm s}\partial _xB_ - + \left( {\delta _c + \delta - k_{\rm s}^2} \right)B_ - = - \phi _ - - \beta k_{\rm s}^2B_ + ,$$

8 $$i\partial _t\phi _ + + \left( {\delta - \left| {\phi _ + } \right|^2 - g\left| {\phi _ - } \right|^2} \right)\phi _ + = - B_ + ,$$

9 $$i\partial _t\phi _ - + \left( {\delta - \left| {\phi _ - } \right|^2 - g\left| {\phi _ + } \right|^2} \right)\phi _ - = - B_ - .$$

The linear dispersion of the ground-state polariton modes given by these equations is shown with the dotted lines in Fig. 2. There is no change of sign of the polariton mass in this approximation and therefore non-radiating solitons should indeed exist. Also, close to *k*_s_ the dispersion in the approximate model matches the exact, thereby justifying the approximations made.

The exact soliton solutions of Eqs. (6)–(9) are sought as exponentially localised pulses moving with group velocity *v*:

10 $$B_ \pm (t,x) = b_ \pm (\xi ),\phi _ \pm (t,x) = \varphi _ \pm (\xi ),\xi = x - vt.$$

**Fig. 3:**
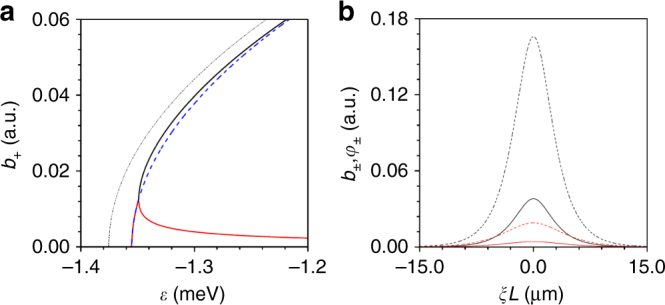
Soliton branches and soliton profiles. **a** Soliton amplitudes for the TM (thin dash-dotted line), TE (blue dashed line) and *σ*_±_ (solid red and black lines) families vs. the energy offset *ε* = *δħ*Ω_*R*_ for *v* = 0.15, *k*_s_ = 1.5, *β* = 0.02. The linear TE-TM energy splitting, 20 μeV, can be seen at *b*_+_ = 0, which is consistent with the experimental data (Supplementary Figs. 1 and 2). **b** Spatial profiles of the photonic (solid lines) and excitonic (dashed lines) components of the *σ*_+_ soliton for $\epsilon$ = −1.305 meV (*δ* = −0.29). Black lines in **b** show *b*_+_, *φ*_+_, while red lines show *b*_−_, *φ*_−_

Solving for *b*_±_(*ξ*), *φ*_±_(*ξ*) numerically we have found two families of linearly polarised solitons, such that *b*_+_ = *b*_−_ (TM-solitons) and *b*_+_ = −*b*_−_ (TE-solitons) (Fig. 3). Note that since *v* ≠ 0, the soliton spectral centre of mass is appreciably shifted away from *k*_s_. A branch of elliptically polarised solitons splits from the low amplitude TE family at some critical value of *δ* (Fig. 3a), which is determined by the spin-orbit coupling parameter *β*. This branch is doubly degenerate, which follows from the symmetry (*B*_+_, *B*_−_, *ϕ*_+_, *ϕ*_−_) → (*B*_−_, *B*_+_, *ϕ*_−_, *ϕ*_+_) of Eqs. (6)–(9), and consists of the elliptical right- and left-polarised solitons, which are referred to below as *σ*_+_ and *σ*_−_ families. The polarisation ellipse of these families is elongated along the TE direction. An example of the spatial profile of the *σ*_+_ soliton is shown in Fig. 3b.

To study the stability of the solitons we first linearised Eqs. (6)–(9) assuming that the solitons, Eq. (10), are perturbed by small-amplitude localised perturbations evolving in time as *e* ^*λt*^. We then solved the linear eigenvalue problem for the perturbations numerically. We have found that TM-solitons are unstable in their entire existence domain and can transform during their evolution into a pair of elliptically polarised quasi-solitons. TE-solitons are only stable before the boundary at which the *σ*_±_ families split from them (Fig. 3a). Note that as we found numerically, the sign of the nonlinear cross-spin interaction, *g*, is critical for maintaining stability of TE-solitons. For the small negative value of *g* we use here TE-solitons are stable in the range discussed above while TM-solitons are unstable. The situation is reversed if *g* has the opposite sign. Instability growth rates *λ*Ω_*R*_ for both families typically reach the values ~1 ps^−1^ in physical units. In contrast, the *σ*_±_-soliton families are unstable only in a very narrow negligible interval of energies adjacent to their bifurcation point and are stable otherwise. This analysis suggests choosing the TE orientation of the input pulses in modelling of the full system and in the experiments, to minimise the complexity of the nonlinear dynamics and facilitate the faster and cleaner convergence of the input to quasi-solitons.

### Polariton Cherenkov radiation

A soliton is, by definition, a wavepacket with suppressed group velocity dispersion, implying that the energy-momentum (dispersion) characteristic of the soliton spectrum is a straight line, the second derivative of which is zero (Fig. 2). Repulsive nonlinearity increases the energy of the nonlinear polariton wave leading to the so-called blue shift. Therefore, the soliton dispersion is shifted upwards from the linear spectrum. The tilt of the line gives the soliton group velocity. Any intersection of the soliton dispersion with the energy spectrum of the linear polaritons gives a resonance momentum, such that the soliton is expected to emit a dispersive wavepacket with the spectrum centred at the resonance momentum.

Dispersion of the TE and TM ground-state modes in our system can be well approximated by the following expressions:

11 $$\delta _{{\mathrm{TE,TM}}} = - \frac{1}{2}\left( {\delta _c - (1 \pm \beta )k^2} \right) - \sqrt {\frac{1}{4}\left( {\delta _c - (1 \pm \beta )k^2} \right)^2 + 1} .$$

Plus and minus signs correspond to the TE and TM modes, respectively. The straight line soliton dispersion is given by *δ*_+_ *v*(*k* − *k*_s_). Hence the Cherenkov resonance conditions are

12 $$\delta + v\left( {k - k_s} \right) = \delta _{{\mathrm{TE,TM}}}.$$

For mathematical details of the derivation of Eqs. (11) and (12) see Supplementary Note 1. Graphical solutions of Eq. (12) are shown in Fig. 2. Depending on the soliton momentum, the momentum and group velocity of the Cherenkov radiation are either close to zero or negative, i.e., the radiation either stays close to where it was emitted inside the waveguide or propagates backwards. Note, the choice of the TE-TM splitting parameter *β* has very little influence on the location of the Cherenkov resonances for experimentally realistic values (Fig. 2). The backward character of the polariton dispersive radiation is a distinct feature of the quasi-soliton dynamics in MCWs, which contrasts it with the forward propagating Cherenkov dispersive waves in optical fibres^11^ or silicon waveguides^14^, for example. This backward radiation was found to be very pronounced in the experiments, since it naturally separates from the forward propagating wavepacket.

### Modelling of the nonlinear pulse propagation in MCWs

**Fig. 4:**
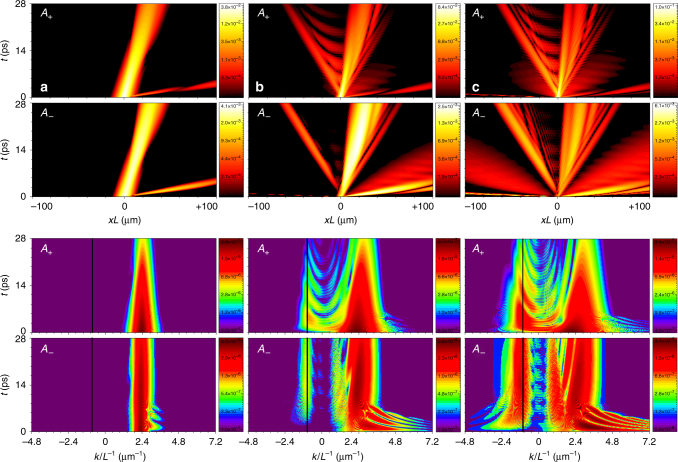
Emission of the backward Cherenkov radiation. Results of the 2D modelling of Eqs. (1)–(4) showing evolution of the quasi-solitons and of the backward-emitted Cherenkov radiation with the negative momentum and negative group velocity. The parameters are *δ* = −0.29 (*ε* = −1.305 meV) (column **a**), *δ* = −0.24 (*ε* = −1.08 meV) (column **b**), *δ* = −0.2 (*ε* = −0.9 meV) (column **c**), *γ*_*c*,*e*_ = 0.01. (*x*, *t*)-plots show the space-time dynamics of |*A*_±_(*x*, *t*)| and (*k*, *t*)-plots show the corresponding evolution of the photon density in the momentum space, all are for *y* = 0. The waves deviating towards negative values of *x* in the (*x*, *t*)-plots in columns **b** and **c** are the backward Cherenkov radiation. Vertical black lines on the (*k*, *t*)-plots show theoretically predicted momenta of the Cherenkov radiation

In order to see how Cherenkov radiation is emitted in the realistic 2D geometry and with practical losses present, we have carried out a series of numerical simulations of Eqs. (1)–(4), initialising them with the quasi-soliton pulses. Selected results of these simulations are shown in Fig. 4 and demonstrate the spatial and spectral dynamics of the pulses. We have tuned the central momentum of the input pulses to 2.4 μm^−1^, as in the experiments described below. The corresponding Cherenkov resonance calculated using Eq. (12) appears at the momentum −0.9 μm^−1^ (Fig. 2), and hence corresponds to the backward radiation. We scanned the energy offset parameter $\epsilon$ = *ħ*Ω_*R*_ *δ* starting from the lower polariton branch at the soliton momentum and into the continuum spectrum (Fig. 2). This scan corresponds to the increase of the input pulse amplitude and power (Fig. 3a). For the relatively small $\epsilon$, see the column a in Fig. 4, we observed metastable propagation of the *σ*_±_ quasi-solitons, which decay adiabatically due to losses and do not emit any significant radiation, as can be seen on the momentum space evolution plots. Column b in Fig. 4 shows the dynamics of the *σ*_+_ quasi-soliton for larger energies. The pulse propagation in this regime is accompanied by the emission of the backward Cherenkov radiation with negative momentum, which matches the predictions of Eq. (12).

Note, a narrow radiation wavepacket on the space-domain plots emitted with a large positive momentum. This effect is not covered by Eq. (12). However, it is unavoidable because the soliton dispersion crosses the shaded continuum at the high momenta (Fig. 2). Note that the signal at these momenta was not measured in our experiments discussed below. With further increase of the initial pulse power (column c in Fig. 4), the number of polaritons transferred into the backward radiation becomes comparable to the number of polaritons remaining inside the deteriorating quasi-soliton pulses. The soliton remnants and the radiation wavepacket spread out, overlap and produce a complex interference pattern. The role of the polariton continuum at large positive momenta becomes especially pronounced in this regime.

### Experimental measurements

For the experiments we used a high-quality microcavity of length $\frac{{3\lambda }}{2}$ grown by molecular beam epitaxy and containing three InGaAs quantum wells (10 nm thick, 4% indium). The DBR mirrors are GaAs/AlGaAs (85% Al) with 26/23 repeats on the bottom/top mirror, respectively. The microcavity was previously described in ref. ^51^. The detuning between the exciton and the photon modes is ≃− 2 meV. The top mirror was partially etched (down to the last few layers of the DBR), defining 1000 μm (*L*_*x*_) long mesas (MCWs) of different widths, as shown in Fig. 1. Polariton Rabi splitting in this structure is $\hbar \Omega _R \simeq 4.5$ meV and the exciton-polariton lifetime is ≃30 ps. For more details about the polariton dispersion characteristics of the sample see Supplementary Figs. 1 and 2. For all our measurements we used the same 5 μm-wide MCW. Our control parameters were the excitation power, centre wavelength and the angle of the excitation beam along the *x*-axis, which is equivalent to the momentum *k*_*x*_ along the MCW. Here *k*_*x*_ = *k*/*L* as used in the theory above.

**Fig. 5:**
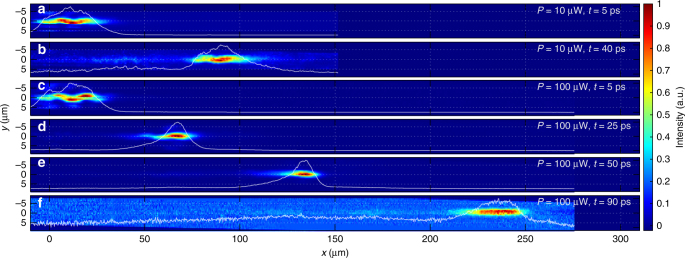
Experimental observations of real-space evolution in time. **a**, **b** Images of the polariton pulse at different times (5 and 40 ps, respectively) with the low excitation power *P* = 10 μW. **c**–**f** Images of the polariton pulse (excitation beam *P* = 100 μW) showing the soliton compression effect (**d**, **e**) followed by the expansion (**f**) captured at 5 ps (**c**), 25 ps (**d**), 50 ps (**e**) and 90 ps (**f**). All images are reconstructed from tomographic scans performed using the streak camera. Time *t* = 0 corresponds to the arrival of the excitation pulse. White lines show the normalised intensity profiles integrated over *y*

First, we applied the ≃5 ps-long, full width at half maximum (FWHM), TE-linearly polarised excitation pulse at $k_x \simeq 2.2$ μm^−1^, above the point of inflection of the lower polariton branch. In this way we excited polariton pulses in the negative effective mass region, which is expected to favour formation of bright solitons. By varying the power of the excitation beam we were able to observe two types of pulse behaviour: quasi-linear propagation at low powers and solitonic compression at higher powers (Fig. 5). The excitation beam creates a pulse with a width of ≃30 μm in the propagation direction *x*, and couples mostly to the ground polariton mode and somewhat less efficiently to the first excited state of the MCW. The superposition of these two modes results in a snake-like pattern in real space as can be seen on Fig. 5a, c. At the low power *P* = 10 μW the pulse propagates until the photoluminescence signal decays to the noise level (Fig. 5a, b). The pulse also extends in real-space changing its shape with time through the group velocity dispersion.

**Fig. 6:**
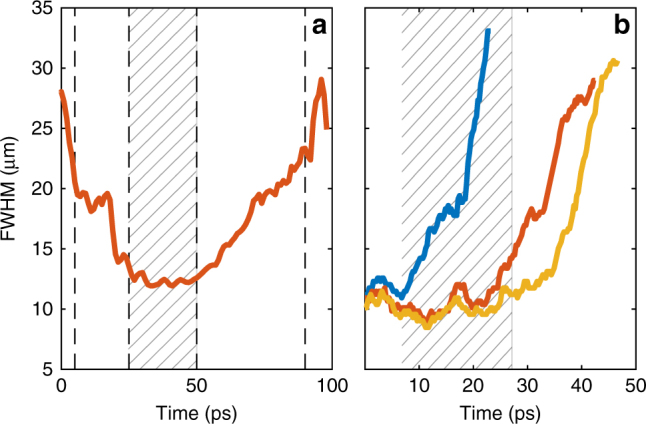
Soliton compression effect. **a** Five-picosecond excitation pulse, *P* = 100 μW. FWHM vs. time, showing the initial threefold compression and the subsequent expansion of the pulse. Black dashed vertical lines indicate times (5, 25, 50 and 90 ps) corresponding to the real-space images shown in Fig. 5c–f. **b** Two-picosecond excitation pulse. FWHM vs. time at different excitation powers (blue: *P* = 30 μW; red: *P* = 250 μW; yellow: *P* = 400 μW). Time *t* = 0 corresponds to the excitation pulse arrival

In contrast, at a higher power, *P* = 100 μW, we observe a significant shrinking of the pulse width to ≃12 μm during the first 40 ps (Figs. 5d, e and 6a). This indicates that the input conditions are such that the pulse achieves the balance between the opposing dispersion and nonlinearity-induced chirps needed for the formation of bright solitons. Figure 6a details the pulse width variation with time in this regime. Up to about 40 ps the pulse undergoes a strong compression. The soliton compression effect for initially unchirped pulses is well known in nonlinear optical waveguides and fibres, see, e.g., refs ^52,53^. After the point of maximal compression, the pulse may either start spreading out or can break-up into a train of less intense solitons, so-called soliton fission^11^. In our case the power vs. loss balance is such that the compression is followed by adiabatic expansion. Thus, this set of measurements confirms the existence of the quasi-soliton pulses in the regime, when their Cherenkov radiation either does not appear or negligible.

In a complementary experiment, also confirming the soliton formation, we excited polariton wavepackets with a spot size (FWHM) of ≃11 μm using a 2-ps pump pulse. The pump momentum was chosen slightly higher, at $k_p \simeq 2.4$ μm^−1^, which is expected to favour emission of the backward Cherenkov radiation. In this case at the lowest power, 30 μW, when the nonlinearity is negligible, the FWHM of the excited wavepacket quickly increases up to 30 μm due to the strong polariton dispersion (Fig. 6b). By contrast, as seen on Fig. 6b, at higher powers, 250 and 400 μW, the polariton-polariton interaction compensates dispersion leading to the non-dispersive propagation for about 30 ps, when the pulse width fluctuates only slightly between 8 and 12 μm. This measurement provides a clear evidence for the bright soliton formation. At longer times (>30 ps) the interplay between polariton losses and dispersion again results in broadening of the excited soliton with time. We note that in our system the polariton-polariton interaction is so strong that it enables solitons containing only 200–300 polaritons at the threshold.

**Fig. 7:**
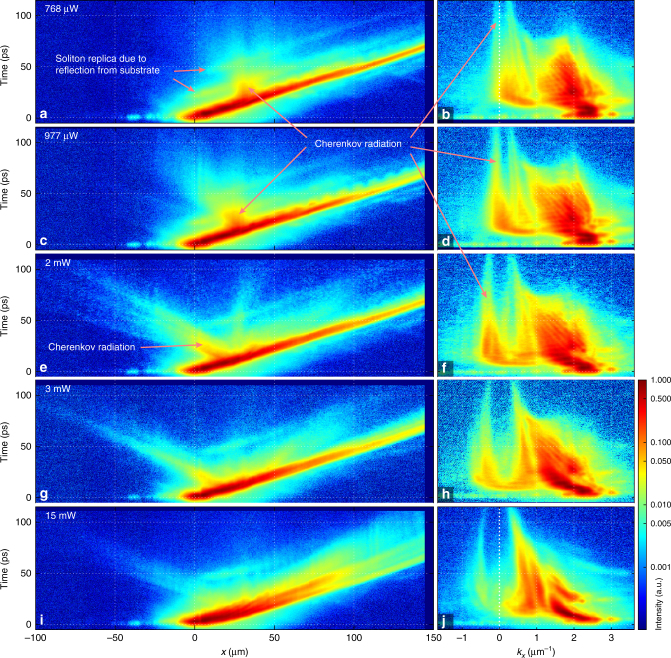
Experimental data showing the backward Cherenkov radiation. **a**, **c**, **e**, **g**, **i** Polariton emission intensity vs. time and the propagation direction *x* measured at the centre of the MCW (*y* = 0 ± 0.2 μm) for the increasing excitation beam powers. Time *t* = 0 corresponds to the excitation pulse arrival. Bright red right-tilted spation-temporal traces show the input pulses and their subsequent splitting into multiple pulses. Paler left-tilted radiation patterns correspond to the Cherenkov radiation propagating in the backward direction. Reflections of the soliton emission from the the polished surface of our sample substrate leads to the appearance of weak soliton replica delayed with respect to the main soliton emission by ~20 and ~40 ps. **b**, **d**, **f**, **h**, **j** The corresponding polariton emission vs. time and *k*_*x*_ momentum recorded at $k_y \simeq 0$ for different excitation powers. All plots use the same logarithmic pseudo-colour map

The Cherenkov resonance conditions, see Eq. (12), predict that the momentum of the radiation will be very sensitive to any change in excitation power or momentum through parameters *δ*, *k*_s_ and *v* (Figs. 2 and 3a). At a lower power the line of the soliton dispersion shifts closer to the dispersion of linear polaritons and we expect Cherenkov emission to shift closer to *k*_*x*_ = 0, while at a higher power it is expected to shift more towards negative momenta in the interval from 0 to −2 μm^−1^. We recorded soliton traces in both *x* coordinate-time and *k*_*x*_ momentum-time spaces for different excitation powers well above the soliton formation threshold (Fig. 7). The pump spot size, pump momentum and energy were chosen to be the same as used to obtain the data shown in Fig. 6b.

At $P_{cr}^{th} \simeq 770$ μW, Cherenkov radiation appears at $k_{cr} \simeq 0$ at ≃20 ps as a nonpropagating polariton emission on Fig. 7a. The corresponding soliton spectrum in momentum space, shown on Fig. 7b, consists of a broad range of harmonics with different *k*-vectors populated by polariton-polariton scattering^32,35^ from the initially excited state, thus triggering the Cherenkov radiation. With an increase of the input power to 977 mW, as expected, the Cherenkov radiation starts propagating backwards with negative momentum ≃−0.2 μm^−1^ (Fig. 7c, d). The momentum of the Cherenkov radiation and hence the negative group velocity gradually increase from 0 at *P* = 770 μW to ≃−0.5 μm at *P* = 15 mW leading to the fast backward-propagating Cherenkov emission in real space (Fig. 7).

Finally, at higher excitation powers the emergence of the solitonic Cherenkov radiation is accompanied by a complex dynamics of the soliton pulse involving dynamics reminiscent of the soliton fission in optical fibres^11^. At the input power *P* = 3 mW (Fig. 7g), the input pulse splits into a pair with a weak second peak appearing at ≃50 ps. At *P* = 15 mW we observe formation of a more complex multi-peak structure. In this regime the emission spectrum in momentum space also becomes modulated (Fig. 7j). We note, that at 2 and 3 mW there are wavepackets, which propagate forward with a velocity smaller than that of the main soliton (Fig. 7e, g). They appear 5–10 ps after the onset of the backward Cherenkov radiation. These structures can be also associated with the forward Cherenkov emission by the evolving soliton due to gradual photonic losses of polaritons. At higher powers, 2–15 mW, the central wavevector and width of the momentum distribution gradually decrease with time (Fig. 7f, h, j). It is well known that polaritons undergo multiple polariton-polariton, polariton-phonon and polariton-free-carrier scattering, which relaxes their momentum towards zero^42^, accounting for the observations in Fig. 7f, h, j. The group velocity in the space-time maps (left column in Fig. 7) does not visibly change because the central momentum is near the point of inflection, where the velocity is close to independent of momentum.

Overall, the spatio-temporal traces in both real and momentum space are in good qualitative agreement with the numerical results on Fig. 4, where one can see similar time domain dynamics and spectral evolution. The latter features the pronounced backward Cherenkov radiation at similar momenta and gradual decay of the quasi-soliton and radiation with time due to losses.

## Discussion

We have considered theoretically and experimentally the nonlinear effects accompanying ultra-short pulse propagation in a pump-free MCW guiding exciton-polaritons. We have introduced an approximate model that has exact soliton solutions and demonstrated the existence of the energy-momentum matching between the soliton and linear polariton waves resulting in the existence of the backward Cherenkov radiation emitted by the polariton solitons. We have experimentally measured nonlinear pulse compression, formation of quasi-solitons and emission of the intense backward Cherenkov radiation. In particular, we have observed that the quasi-solitons are formed without noticeable Cherenkov radiation for relatively low input powers and that the power increase above threshold triggers pronounced backward radiation. In this work we have studied a one-dimensional micro-wire and demonstrated the fundamental process of backward Cherenkov radiation by one-dimensional solitons. We anticipate a host of new polariton radiation physics in a system without lateral confinement, where the higher dimensionality and anisotropy of the polariton mass adds a wealth of additional effects, see, e.g., refs ^54,55^. The experimentally measured and numerically modelled spatio-temporal patterns showing formation of the quasi-solitons and the backward Cherenkov radiation are in excellent qualitative agreement. MCWs are considered to be building blocks of future all-polariton information processing circuits^26^, where the soliton and Cherenkov radiation effects described in this work may be expected to play an important role, as they already do in conventional semiconductor nanophotonics^12–16^ and fibre optics^10,11,17^.

### Data availability

The data that support the findings of this study are available upon reasonable request sent to the corresponding author.

## Electronic supplementary material

Supplementary Information
