# Supplementary material for: Backward Cherenkov radiation emitted by polariton solitons in a microcavity wire
Source: Nat Commun. 2017 Nov 16;8:1554. doi: 10.1038/s41467-017-01751-6 (PMC5691178; doi:10.1038/s41467-017-01751-6)
Supplement: Supplementary file 1 — Supplementary Information [file 41467_2017_1751_MOESM1_ESM.pdf]

SUPPLEMENTARY NOTE 1: DERIVATION OF THE PHASE MATCHING CONDITION FOR THE BACKWARD CHERENKOV RADIATION

A soliton is, by definition, a wavepacket with suppressed group velocity dispersion, implying that the energy-momentum (dispersion) characteristic of the soliton spectrum is a straight line, the second derivative of which is zero. Repulsive nonlinearity increases the energy of the nonlinear wave. Therefore, this line is shifted upwards from the linear spectrum. The tilt of the line gives the soliton group velocity. Any intersection of the soliton dispersion with the energy spectrum of the linear polaritons gives a resonance momentum, such that the soliton is expected to emit a dispersive wave packet with the spectrum centred at the resonance momentum, see Fig. 2.

In order to formalize this concept we use the anzats containing the exact soliton solutions, $b_\pm$, $\varphi_\pm$, and the radiation field $\varepsilon_\pm$, $\alpha_\pm$:

$$A_\pm(t,x) = b_\pm(\xi)e^{ik_s x} + \varepsilon_\pm(\xi,t)e^{i\delta t}, \quad (1)$$
$$\psi_\pm(t,x) = \varphi_\pm(\xi)e^{ik_s x} + \alpha_\pm(\xi,t)e^{i\delta t}. \quad (2)$$

We substitute Supplementary Equations (1) and (2) into the 1D approximation of Eqs. (1) with $\partial_y = 0$, where $\gamma_{c,e} = \partial_y = U(y) = 0$, and linearise around $\varepsilon_\pm$, $\alpha_\pm$. The resulting equations for the amplitudes of the radiation fields

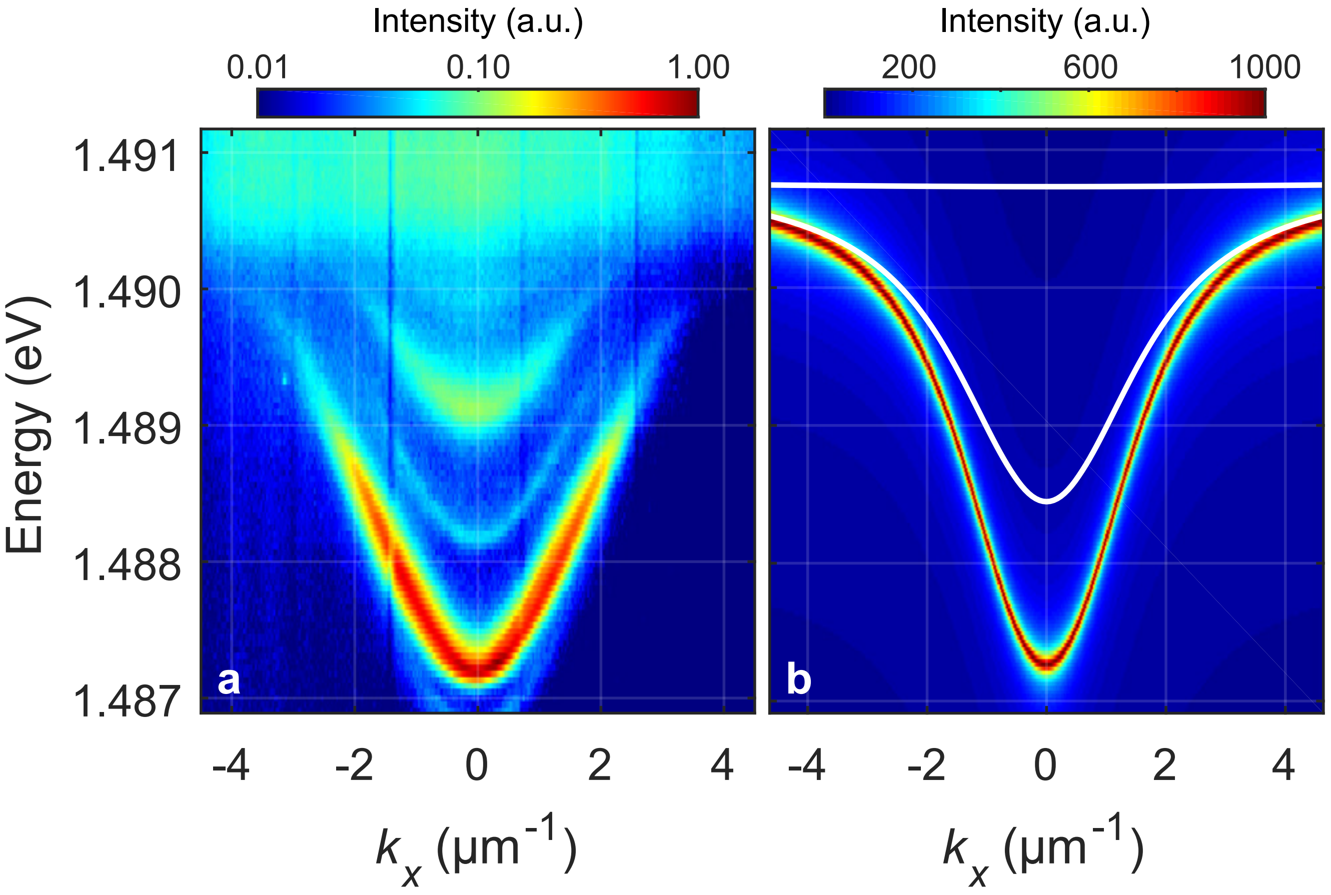

Supplementary Fig. 1. Polariton dispersion. (a) Experimentally measured polariton dispersion in the microcavity wire. The brightest line correspond to the ground state and the next line up to the first excited state. (b) Numerically computed polariton dispersion accounting for the polariton lifetime. Continuous spectrum is located between the white lines.

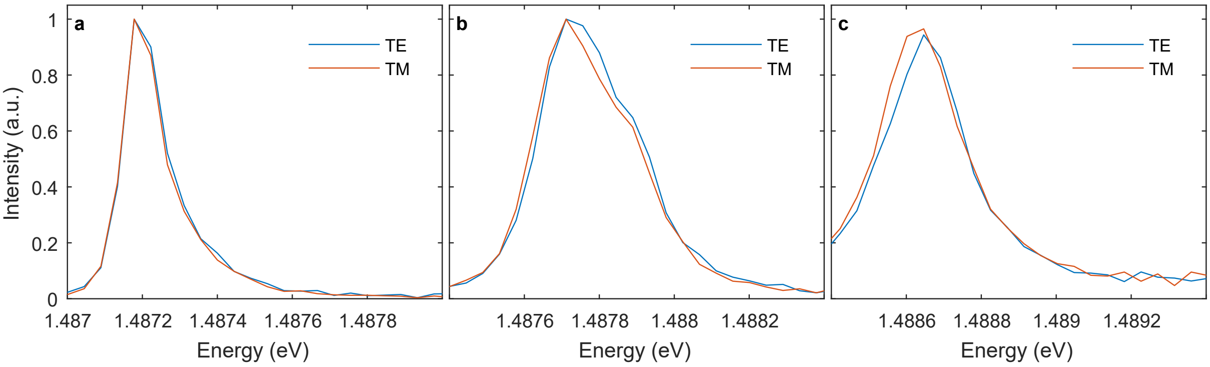

Supplementary Fig. 2. TE-TM energy splitting. Intensity of the TE and TM polarizations vs photon energy at $k_y \simeq 0$ and for different values of k_x: (a) $k_x \simeq 0$, (b) $k_x \simeq 1$ μm^{-1}, and (c) $k_x \simeq 2$ μm^{-1}.

form a system of the linear inhomogeneous partial differential equations:

$$i\partial_t \varepsilon_+ + \partial_x^2 \varepsilon_+ + \delta_c \varepsilon_+ + \alpha_+ - \beta \partial_x^2 \varepsilon_- = e^{ik_s x - i\delta t}(-\partial_x^2 b_+ + \beta 2k_s \partial_x b_-) \tag{3}$$

$$i\partial_t \varepsilon_- + \partial_x^2 \varepsilon_- + \delta_c \varepsilon_- + \alpha_- - \beta \partial_x^2 \varepsilon_+ = e^{ik_s x - i\delta t}(-\partial_x^2 b_- + \beta 2k_s \partial_x b_+) \tag{4}$$

$$i\partial_t \alpha_+ + \varepsilon_+ = 0 \tag{5}$$

$$i\partial_t \alpha_- + \varepsilon_- = 0. \tag{6}$$

The operators on the left hand-sides of Supplementary Equations (3)-(6) describe dispersion of the linear polaritons while the right-hand sides represent the source term which is pumping energy into the Cherenkov radiation. The origin of the source term is the mismatch between the full polariton dispersion and the approximate dispersion that ensures the existence of the exact solitons. The terms $\beta\partial_x^2 b_\pm$ are neglected since they are an order or more smaller than the other two terms in the right-hand sides of Supplementary Equations (3), (4). In deriving Supplementary Equations (3)-(6), we retained the perturbation terms linear in the soliton amplitude and disregarded the nonlinear ones.

In order to reveal the solution structure of Supplementary Equations (3)-(6), we write them in the matrix form $i\partial_t \mathbf{r} + \hat{L}(\partial_x^2)\mathbf{r} = e^{ik_s x - i\delta t}\mathbf{R}(x - vt)$, where $\mathbf{r} = (\varepsilon_+, \varepsilon_-, \alpha_+, \alpha_-)^T$, $\mathbf{R}$ is the right-hand side of Supplementary Equations (3)-(4) and $\hat{L}$ is the 4×4 matrix operator with the structure evident from the left-hand side of Supplementary Equations (3)-(4). Replacing the radiation, $\mathbf{r}$, and the source, $\mathbf{R}$, vectors with their integral Fourier expansions: $\mathbf{r}(x,t) = \int_{-\infty}^{\infty} dk \mathbf{r}_k(t) e^{ikx}$, $\mathbf{R}\ e^{ik_s x} = \int_{-\infty}^{\infty} dk' \mathbf{R}_{k'} e^{i(k'+k_s)x - ik'vt} = \int_{-\infty}^{\infty} dk \mathbf{R}_{k-k_s} e^{ikx - i(k-k_s)vt}$, we find:

$$i\partial_t \mathbf{r}_k + \hat{L}(-k^2)\mathbf{r}_k = e^{-i(kv - k_s v + \delta)t}\mathbf{R}_{k-k_s}. \tag{7}$$

Under the assumption that $\mathbf{r}_k \sim e^{-i\delta' t}$, the eigenfrequencies of the operator on the left-hand side are found from $\det(\delta' + \hat{L}(-k^2)) = 0$, and they are naturally the frequencies of the TE and TM ground state modes:

$$\delta_{\mathrm{TE,TM}} = -\frac{1}{2}(\delta_c - (1 \pm \beta)k^2) - \sqrt{\frac{1}{4}(\delta_c - (1 \pm \beta)k^2)^2 + 1}. \tag{8}$$

The minus sign before the square root corresponds to the lower polariton branch. The upper polariton branch does not introduce new resonance conditions. The solitonic source term oscillates with the net frequency $\delta + v(k - k_s)$, see Supplementary Equation (7), hence conditions providing the resonances between the linear spectrum and solitons are

$$\delta + v(k - k_s) = \delta_{\mathrm{TE,TM}}. \tag{9}$$

SUPPLEMENTARY NOTE 2: SAMPLE CHARACTERISATION

Supplementary Figure 1(a) shows the polarization unresolved dispersions of different modes associated with the lower polariton branch. The brightest line corresponds to the ground state mode. SI Fig. 1(b) shows numerically computed TE ground state dispersion that accounts for the finite polariton lifetime, while Fig. 2 in the main text assumes the infinite lifetime.

Supplementary Figure 2 shows polariton PL emission spectra detected for three values of the momentum in both TE and TM polarisations. The splitting varies with momentum k_x. At $k_x = 0$ it is close to 0 and at 2 μm^{-1} it is about $20 - 30$ μ eV. We have chosen the value of 20μeV, corresponding to $\beta = 0.02$, in our numerical modelling, see Fig. 3(a).
